# Iatrogenic esophageal perforation that could be treated indirectly by cervical esophagostomy and laparoscopic surgery

**DOI:** 10.1016/j.ijscr.2019.05.053

**Published:** 2019-06-03

**Authors:** Ryohei Matsui, Satoru Takayama, Taku Hattori, Toru Imagami, Masaki Sakamoto, Hisanori Kani

**Affiliations:** Department of Surgery, Nagoya Tokushukai General Hospital, Kasugai, Japan

**Keywords:** AS, aortic valve stenosis, TEE, transesophageal echocardiography, CT, computed tomography, Case report, Esophageal perforation, Esophagostomy, Laparoscopic surgery, Two-stage surgery

## Abstract

**Introduction:**

Successful nonoperative management has been reported for esophageal perforation; however, some cases require surgery.

**Case presentation:**

We presented the case of an 85-year-old woman with iatrogenic thoracic esophageal perforation in whom primary repair or resection of the perforated esophagus was difficult because she was elderly and had severe aortic valve stenosis. Therefore, we selected a two-stage surgery; laparoscopic gastrostomy, jejunostomy, posterior mediastinal drainage, and cervical esophagostomy were performed. We planned reconstruction after the perforation was closed, but endoscopic examination revealed spontaneous patency of each esophageal stump. Endoscopic balloon dilation was necessary because of esophageal stenosis; however, anastomotic surgery was unnecessary.

**Conclusion:**

This case report suggests that esophageal perforation is resolved without direct closure if appropriate drainage is performed.

## Highlights

• It is very rare case that each esophageal stump become connected and patent spontaneously.
• Two-stage surgery is useful for esophageal perforation if radical operation is difficult.
• Esophageal perforation can be resolved without direct closure if appropriate drainage is performed.

## 1 Introduction

Esophageal perforation, a rare disease with high mortality, has various etiologies, such as iatrogenic, idiopathic, trauma, and foreign body; its treatment choice depends on the facility. Although successful conservative therapy has been reported, some cases require surgery. Two-stage surgery and indirect approach may be selected based on patients’ condition, but reconstruction will be necessary after first stabilizing patients from critical situations. Here, we reported a rare case in which esophageal perforation was resolved without direct closure and each esophageal stump achieved spontaneous patency after cervical esophagostomy using a tube. The following case was written in line with the SCARE criteria [1].

## 2 Case presentation

An 85-year-old woman with severe aortic valve stenosis (AS) was admitted to undergo transcatheter aortic valve implantation. She had a history of cerebral infraction, with no remarkable family history. Recently, she experienced chest pain, clammy sweat, and anorexia; she visited a local doctor for AS treatment.

**Fig. 1:**
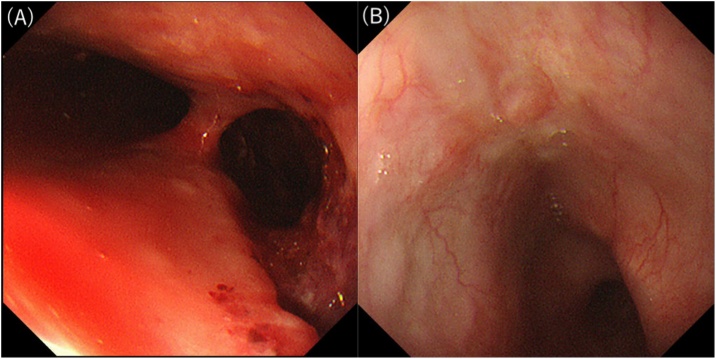
Comparative pictures of the perforation before and after closure. A. Upper gastrointestinal endoscopy reveals a large esophageal perforation located 30 cm from the incisors after TEE. B. The perforation is completely closed at 22 days postoperatively.

**Fig. 2:**
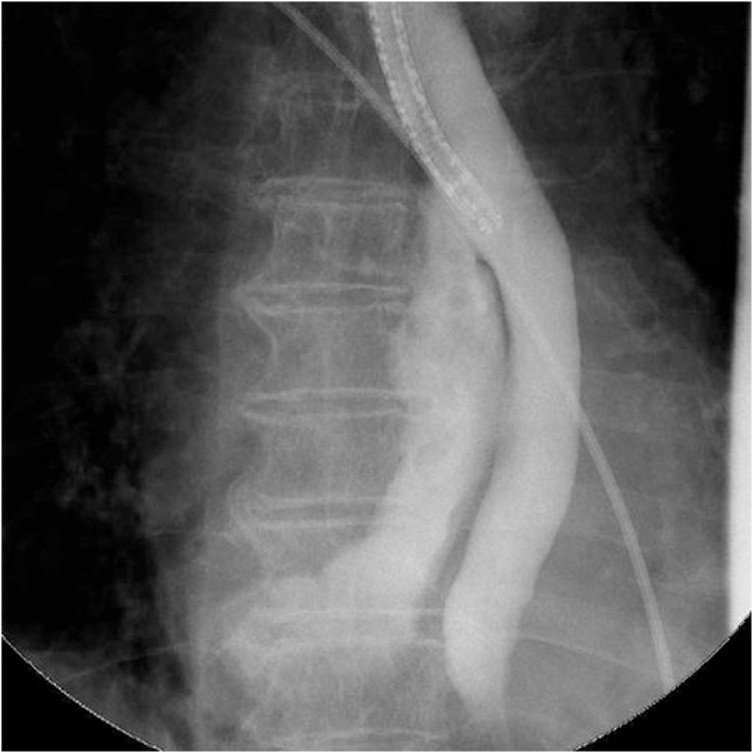
Esophageal contrast with gastrografin confirms remarkable leakage into the mediastinum.

She complained of chest and back pain and developed fever after undergoing preoperative transesophageal echocardiography (TEE). The next day, the symptoms did not improve and computed tomography (CT) revealed prominent mediastinal emphysema and pleural effusion. Upper gastrointestinal endoscopy confirmed esophageal perforation located 30 cm from the incisors (Fig. 1A), and gastrografin contrast revealed mediastinum leakage (Fig. 2).

She was diagnosed with thoracic esophageal perforation. Radical thoracotomy surgery (primary repair or resection) was difficult because she was elderly and had severe AS. Therefore, two-stage surgery and indirect approach, comprising cervical esophagostomy to avoid contamination, gastrostomy for decompression, and jejunostomy for nutrition, was adopted. Reconstruction was planned after the mediastinitis and perforation were healed.

An emergency operation was performed 32 h after TEE under general anesthesia; a 12-mm trocar for the laparoscope was placed through the umbilicus, and four 5-mm ports were placed in the left upper, right upper, left middle, and right middle quadrants. We washed the contaminated mediastinum with saline through the esophageal hiatus from the abdominal cavity side and placed the drainage tube in the mediastinum. We then performed gastrostomy and jejunostomy laparoscopically, followed by cervical esophagostomy using a tube. Esophageal dissection was performed by an autosuture device (operation time: 2 h 14 min; blood loss: minimal).

Postoperatively, clinical course was good. At 11 days postoperatively, CT revealed almost complete resolution of the mediastinal air and cavity and the mediastinal drain was removed.

**Fig. 3:**
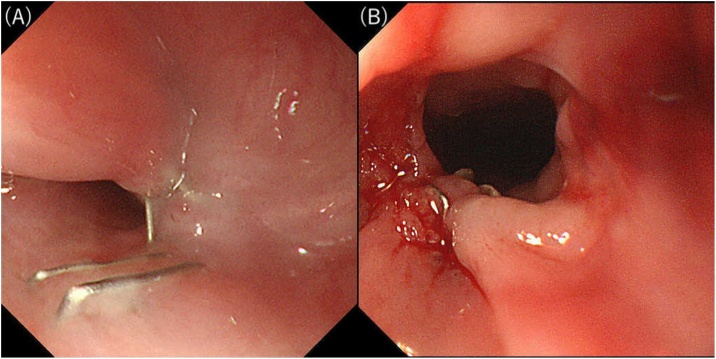
Comparative pictures of the stricture area before and after endoscopic balloon dilatation. A. The cervical esophageal stump separated during the surgery is now connected and patent spontaneously. B. Stricture area after four sessions of dilation.

**Fig. 4:**
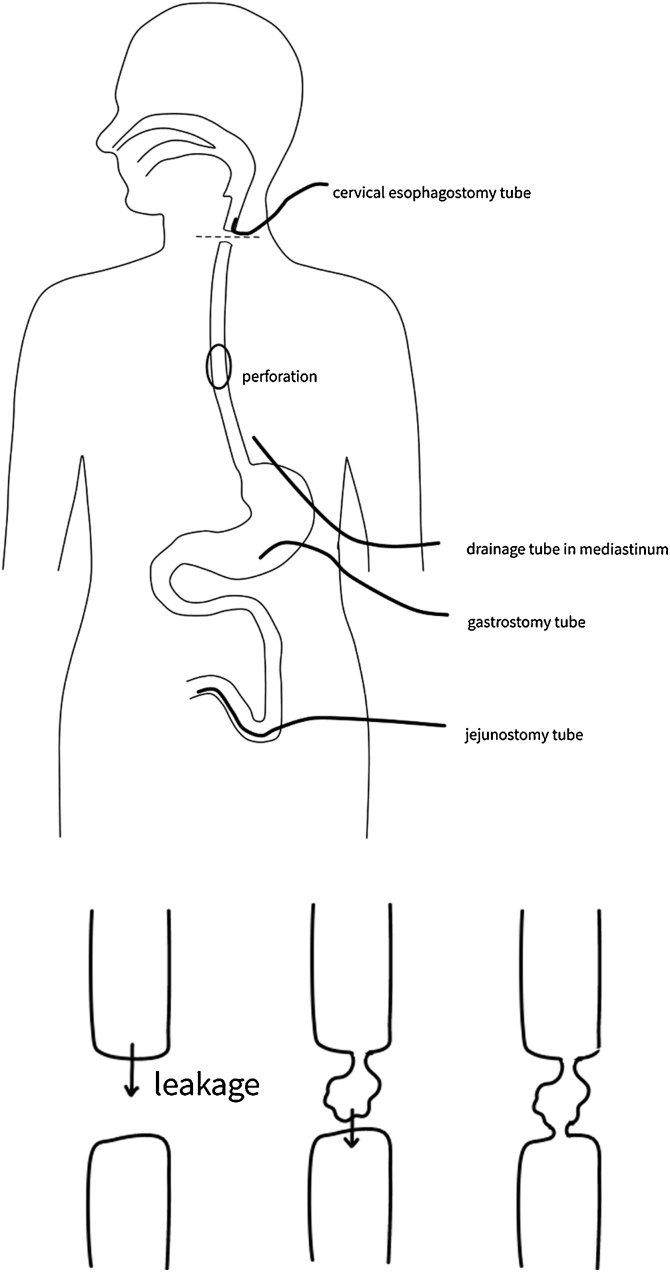
Surgical schema and mechanism of spontaneous patency of each esophageal stump. The oral side of the esophageal stump leaked and caused local contamination; consequently, the anal side was broken and caused each stump to become connected and patent spontaneously.

At 22 days postoperatively, endoscopic retrograde observation via gastrostomy revealed a healed perforation (Fig. 1B), and the cervical esophageal stump that was separated during surgery was connected; it became patent spontaneously (Fig. 3A). Endoscopic findings confirmed that the recanalized segment’s lumen was strong, and we assessed the recanalized segment’s continuity by CT. Anastomotic surgery was unnecessary; however, because of esophageal stenosis, endoscopic balloon dilation was necessary. After four sessions of dilation (Fig. 3B), she could orally consume food without additional surgery. Currently, she can walk with assistance and has a good oral intake (Fig. 4).

## 3 Discussion

Esophageal perforation treatment is based on proper drainage and closure. Conservative treatment or surgery may be selected, depending on the etiology, location of the perforation, time from onset to intervention, and patient’s condition. Conservative treatment mainly involved antibiotics, percutaneous drainage, and nasogastric tube decompression; however, several endoscopic treatments have been recently reported.

Endoscopic clipping has been successfully used widely for esophageal perforation treatment. Lázár et al. summarized the results of esophageal perforation closure with endoscopic clipping [2]. Clips are especially useful for minimally contaminated iatrogenic or spontaneous esophageal perforation. Stent placement has been reportedly effective in many cases [[3], [4], [5]]. Endoscopic stent placement was initially introduced for malignant esophageal stenosis, but it became a widely accepted treatment alternative for anastomotic leaks after esophagectomy and had been adapted for esophageal perforation. Recent reports have described endoscopic vacuum therapy [6,7], during which a nasogastric tube connected to a sponge is endoscopically placed and negative pressure is applied to the lesion. The advantages of this technique include drainage of turbid discharge, improvement of blood flow, reduction of edema, promotion of granulation, and wound closure. Notably, nonoperative management is minimally invasive but cannot be successful without proper selection of cases. Cases with systemic inflammatory response syndrome, pleural effusion, large perforation, or uncontrolled leakage often require operative management [8].

Primary closure of the perforation, with or without tissue buttress, is the most common type of operative management; however, it carries the risk of leakage in late perforation cases [9], for which T-tube drainage may be useful [10]. In some cases, resection of the perforated esophagus may be necessary; however, this is highly invasive and is difficult to perform depending on patients’ conditions. In such patients, cervical esophagostomy and gastrostomy are performed first to prevent further contamination of the perforated lesion, followed by reconstruction [11].

In our patient, the perforation was very large, with a remarkable amount of leakage; therefore, conservative therapy was not indicated. However, the risk of radical thoracotomy surgery was high because of her age and medical history. Therefore, we selected the minimally invasive laparoscopic surgery and cervical esophagostomy. The operation was successfully completed, and the course was good. We planned to perform reconstruction when the perforation was closed; however, endoscopic examination revealed spontaneous patency of the esophagus. The oral side of the esophageal stump leaked and caused local contamination; consequently, the anal side was broken and caused each stump to become connected and patent spontaneously (Fig. 3). Although this finding was unexpected, fortunately, our patient did not require a second operation. The clinical course of this condition is very rare and the patient’s outcome was good. To our knowledge, no such studies have been reported in literature. Taken together, our study suggests that the esophageal perforation is resolved without direct closure if appropriate drainage is performed.

## 4 Conclusion

In patients with esophageal perforation and unstable conditions, two-stage laparoscopic surgery and indirect approach can be useful to minimize invasiveness of management.

## Conflicts of interest

No conflict of interest for all authors.

## Sources of funding

This research did not receive any specific grant from funding agencies in the public, commercial, or not-for-profit sectors.

## Ethical approval

Consent was obtained from the family of the patient. This case report is exempt from ethical approval by our institution.

## Consent

We obtained informed consent from the patient’s family before operation, and written informed consent was obtained from the patient’s family for publication of this case report and accompanying images.

## Author contribution

Each author contributed to diagnosis and treatment. R. Matsui drafted the manuscript. All authors have read and approved the manuscript, and we certify that no portion of this manuscript has been previously published.

## Registration of research studies

This manuscript is not a human study, but a case report.

## Guarantor

Ryohei Matsui

## Provenance and peer review

Not commissioned, externally peer-reviewed
